# Developing a Cell Culture Protocol to Induce Quiescence in Intestinal Fibroblasts Using Design of Experiments Statistical Optimization

**DOI:** 10.21203/rs.3.rs-7677632/v1

**Published:** 2025-10-29

**Authors:** Zahra Mohammadalizadeh, Katherine Bauer Estrada, Morgenne Almonte, Kaitlin C. Fogg, Ana M. Porras

**Affiliations:** University of Florida; Universidad de la Sabana; University of Florida; Oregon State University; University of Florida

**Keywords:** Intestinal fibrosis, fibroblasts, quiescence, activation, extracellular matrix, design of experiments

## Abstract

*In vitro* studies of intestinal fibrosis are confounded by spontaneous fibroblast activation on rigid substrates, hindering the investigation of the early events that initiate fibrosis. To address this challenge, we applied a design of experiments (DOE) framework to systematically evaluate extracellular matrix proteins and soluble factors that influence fibroblast phenotype. Using CCD-18Co colonic fibroblasts, we identified optimized conditions that reproducibly sustained a quiescent state. Validation experiments confirmed that under these conditions, fibroblasts remained spindle-shaped and viable, with reduced αSMA expression and decreased extracellular matrix secretion. These findings demonstrate that DOE can reveal non-linear interactions between microenvironmental cues while enabling the development of a reproducible culture protocol to maintain fibroblast quiescence. This accessible method will allow researchers to investigate the cellular and molecular signals that trigger intestinal fibrosis in a more physiologically relevant context.

## INTRODUCTION

Intestinal fibroblasts are the primary mediators of gastrointestinal fibrosis, orchestrating extracellular matrix (ECM) synthesis and remodeling during tissue repair^1–3^. Under healthy conditions, these fibroblasts remain in a quiescent state but can be activated into myofibroblasts in response to intestinal damage, leading to controlled ECM deposition and wound closure^4,5^. This process is resolved through reversion to a quiescent state or apoptosis^6^. However, in chronic inflammatory conditions like Crohn’s disease, persistent pro-inflammatory cues disrupt this balance. The result is sustained fibroblast activation that drives cycles of matrix degradation and deposition that ultimately result in fibrosis, pathological ECM accumulation^7–9^. Uncontrolled fibrosis can lead to painful strictures and bowel obstructions requiring surgical intervention^10,11^. Furthermore, existing therapies for Crohn’s Disease target inflammation but do not prevent or reverse fibrosis progression^12^. This therapeutic gap is partially driven by an incomplete understanding of the early mechanisms that drive ECM dysregulation and fibroblast activation.

Our ability to investigate early fibroblast activation is hindered by the failure of many *in vitro* models to recapitulate the healthy, quiescent state of intestinal fibroblasts. Typically, colonic fibroblasts are cultured on tissue culture polystyrene (TCPS), a rigid substrate that induces spontaneous fibroblast activation^13–17^. As a result, fibroblasts cultured under these conditions often acquire a protomyofibroblast phenotype, characterized by the formation of stress fibers, even before exposure to additional stimuli^18,19^. These proto-myofibroblasts further differentiate into mature myofibroblasts upon sustained mechanical tension or treatment with soluble profibrotic stimuli like transforming growth factor beta 1 (TGF-β_1_)^8,20–23^. This inherent limitation of conventional culture models prevents the study of the cues that trigger the shift from quiescence to activation, underscoring the need for novel culture systems that support fibroblast quiescence and allow for a more physiologically accurate investigation into the early events leading to intestinal fibrosis.

To address this challenge, we sought to develop a reproducible protocol for inducing and maintaining quiescence in colonic fibroblasts *in vitro*. Building on our prior work inducing quiescence in aortic valve interstitial cells^24^, we hypothesized that specific combinations of ECM coatings and soluble cues could recreate the microenvironmental signals that promote a quiescent fibroblast phenotype. Previous studies have established that both ECM composition and soluble factors regulate fibroblast behavior in the intestines. For example, physiologically relevant ECM proteins - such as collagen types I and III, and laminin - play key roles in shaping fibroblast adhesion, proliferation, and myofibroblastic marker expression^7,25,26^. Soluble factors also shape fibroblast phenotype; vitamin D supplementation has been associated with reduced fibrosis and increased fibroblast quiescence in the colon, both *in vivo* and *in vitro*^27–29^. Similarly, basic fibroblast growth factor (bFGF) promotes intestinal wound healing by modulating fibroblast activation and the secretion of ECM remodeling enzymes^30–32^.

Because testing all potential ECM coatings and soluble factor combinations using traditional one- or two–variable-at-a-time experiments would be both time- and resource-intensive, we employed a Design of Experiments (DOE) framework. DOE is a statistical optimization method that enables the simultaneous evaluation of multiple variables and their interactions, reducing experimental burden while enhancing experimental efficiency, scalability, and reproducibility^33–37^. Here, we used DOE to systematically evaluate combinations of ECM coatings and soluble factors, ultimately identifying the microenvironmental conditions that promote quiescence *in vitro*. Based on these findings, we developed and validated a reproducible protocol for cultivating quiescent colonic fibroblasts *in vitro*.

## RESULTS

### Individual exposure to ECM coatings or soluble factors is insufficient to induce quiescence

**Figure 1:**
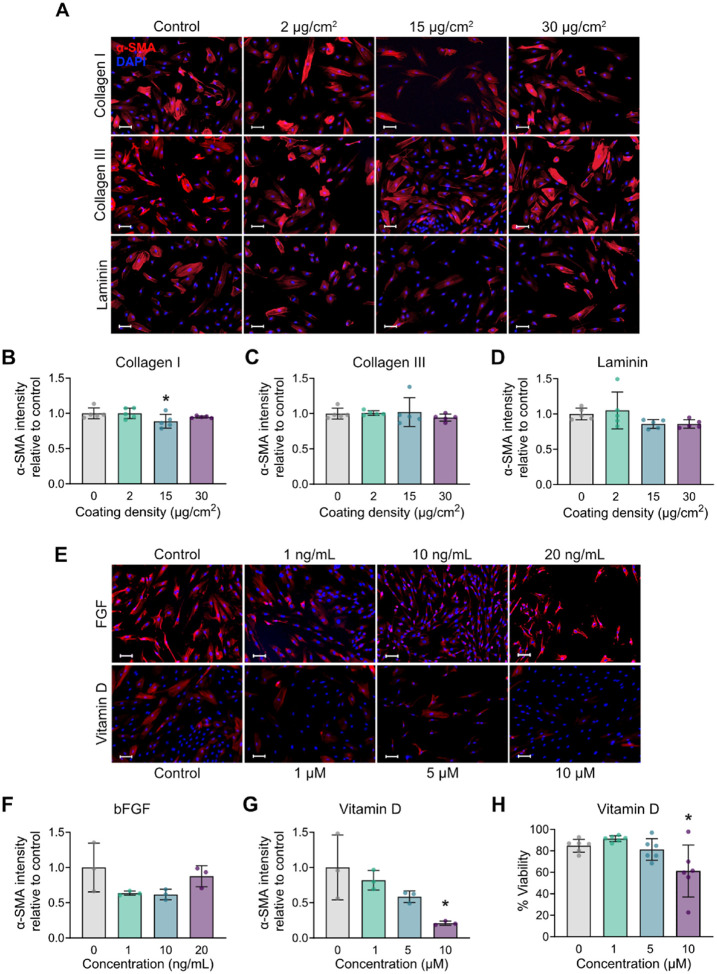
Assessment of the effect of individual factors on fibroblast phenotype. **(A)**Immunocytochemistry images showing α-SMA staining (red) in fibroblasts cultured on collagen I, collagen III, or laminin coatings (2, 15, and 30 μg/cm^2^); nuclei are counterstained with DAPI (blue). **(B–D)** Quantification of α-SMA fluorescence intensity relative to the untreated control for **(B)**collagen I, **(C)** collagen III, and **(D)** laminin. **(E)**Immunocytochemistry images for α-SMA (red) expression in cells treated with FGF (1, 10, 20 ng/mL) or vitamin D (1, 5, 10 μM); nuclei are counterstained with DAPI (blue). **(F–G)** Quantification of α-SMA fluorescence intensity relative to the untreated control for **(F)** FGF and **(G)** vitamin D. **(H)**Quantification of cell viability in response to increasing vitamin D concentrations. **(A-E)** Scale bar indicates 100 μm. n = 3–5 replicates. One-way ANOVA followed by Tukey’s multiple comparisons test. *p < 0.05 compared to the untreated control.

First, we assessed the impact of individual ECM coatings on the expression of α-smooth muscle actin (α-SMA), a canonical marker of myofibroblastic differentiation^8,38^, by CCD-18Co colonic fibroblasts (Fig. 1A). Specifically, we selected collagen I, collagen III, and laminin based on their physiological relevance to colonic tissue. Collagen I and III are the principal fibrillar collagens in the colonic submucosa and muscle layer, where they provide mechanical support and structural integrity that can modulate fibroblast behavior^39,40^. Previous studies have demonstrated that cardiovascular fibroblasts cultured on collagen matrices and coatings can lower the expression of myofibroblastic markers^24,41,42^. Similarly, laminin, a key component of the colonic basement membrane, regulates fibroblast adhesion and proliferation *in vitro*^25,43,44^.

CCD-18Co fibroblasts were cultured for five days in media supplemented with 2% serum on TCPS plates coated with collagen I, collagen III, or laminin at concentrations of 2, 15, or 30 μg/cm^2^. An uncoated TCPS control group was included for baseline comparison. Fibroblasts grown under these control conditions exhibited high levels of α-SMA expression, consistent with an activated phenotype (Fig. 1A).

Culture on the collagen I coatings had a minimal effect on α-SMA expression, with only 15 μg/cm^2^ leading to a modest reduction compared to the control (Fig. 1A–B). In contrast, collagen III had no significant effect on the production of α-SMA at any concentrations (Fig. 1A, 1C). Finally, α-SMA intensity was lower at higher laminin densities (15 and 30 μg/cm^2^); however, these differences were not statistically significant. Collectively, these findings indicate that ECM coatings alone may only partially influence α-SMA expression patterns.

Next, we evaluated whether supplementing the culture medium with vitamin D or human FGF-basic (bFGF)could more strongly influence cell phenotype. Vitamin D has been reported to suppress intestinal myofibroblast activation^27–29^, whereas FGF, a central regulator of cellular migration and survival, exerts context-dependent effects on extracellular matrix dynamics and myofibroblast activation^31,32,45^. For these experiments, CCD18-Co fibroblasts were cultured on TCPS in media containing 2% serum and treated for 5 days with either vitamin D (1, 5, or 10 μM) or bFGF (1, 10, or 20 ng/mL). bFGF treatment did not significantly impact α-SMA expression (Fig. 1E–F). In contrast, vitamin D decreased α-SMA expression in a dose-dependent manner, with the 10 μM concentration showing a statistically significant 5-fold decrease compared to the control (Fig. 1E, 1G). However, further analysis revealed a substantial drop in cell viability at high vitamin D concentrations (Fig. 1H), with more than 40% of cells classified as non-viable at 10 μM. These results suggest that while soluble factors like vitamin D can suppress fibroblast activation, their utility is limited by cytotoxicity at effective doses.

### Identification of Culture Conditions that Promote Fibroblast Quiescence Using DOE

We applied a DOE strategy to investigate whether combinations of ECM coatings and soluble factors more effectively promote fibroblast quiescence than single cues. Specifically, we evaluated the effects of five continuous input variables — collagen I (2–30 μg/cm^2^), collagen III (2–30 μg/cm^2^), laminin (2–30 μg/cm^2^), vitamin D (1–10 μM), and bFGF (1–20 ng/mL) — on four key cellular outcomes: α-SMA intensity, fibronectin secretion, cell viability and proliferation. These outcomes were selected based on their relevance to established phenotypic features of fibroblast activation and quiescence. In the quiescent state, fibroblasts generally display low levels of activation markers like α-SMA^4,42,46^, produce less extracellular matrix proteins such as fibronectin^44,47^, and show reduced proliferative activity^48,49^. Cell viability was also selected as a marker because of the cytotoxic effects of vitamin D observed in the preliminary experiments (Fig. 1H).

We specifically employed a Box–Behnken design resulting in 43 unique experimental conditions (Supplementary Table 1). The control condition consisted of cells seeded on TCPS without coatings and fed media without soluble factor supplementation. For each condition, α-SMA intensity, fibronectin secretion, viability and proliferation were measured after 5 days in culture. All output variables were significantly affected by at least one input variable, with statistically significant linear and second-order interaction effects identified for most responses (Table 1). The ANOVA results also demonstrate that none of the responses exhibited a significant lack of fit (all *p* values > 0.40), indicating that the pure error is small relative to the variation explained by the model (Table 1). All output variables had an R^2^ greater than 0.5, reflecting that the model explains a substantial proportion of the observed variance. Similarly, adjusted R^2^ values above 0.5 confirm that the number of included terms is suitable for the data. The predicted R^2^ values were also within 0.2 of the adjusted R^2^ values, indicating strong consistency between model fit and predictive estimates. Together, these results suggest that the fitted model is appropriate for describing and predicting responses within the studied input factor ranges.

**Figure 2:**
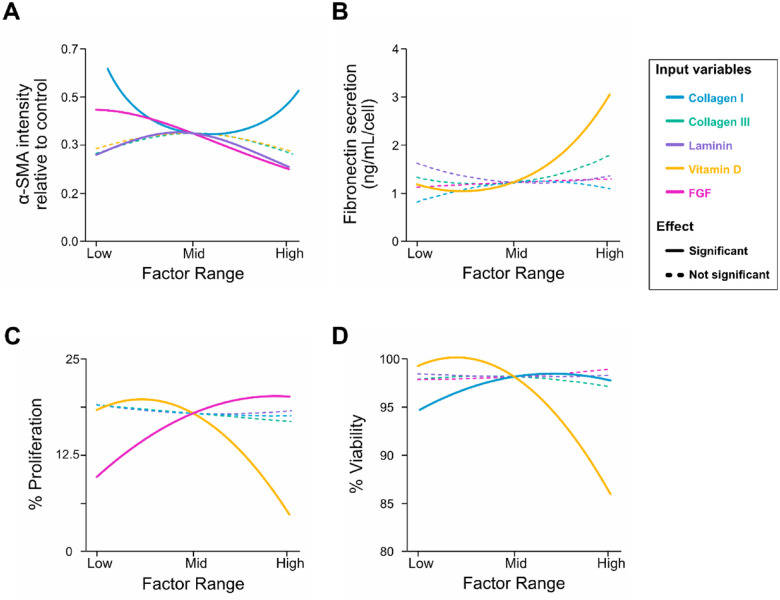
Single-factor effect plots for the four measured responses as a function of the five input variables. **(A)** α-SMA intensity relative to control, **(B)** fibronectin secretion, **(C)** proliferation and **(D)** viability. Plots show the predicted cell responses generated from predictive model equations for each response variable with all other factors held at their midlevel values.Solid lines indicate statistically significant effects, whereas dotted lines represent non-significant effects (Table 1). Quadratic multiple linear regression models for each response were developed using data from 42 experimental conditions, with six replicates per condition.

**Figure 3:**
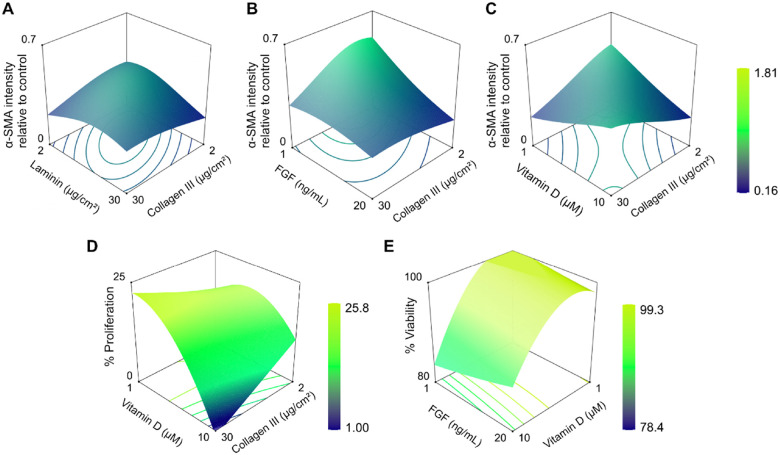
3D response surface plots of model predictions showing significant interaction effects between input variables for each response. Each graph depicts the effect of paired input variables (x- and y-axes) on **(A-C)** α-SMA fluorescence intensity, **(D)** cell proliferation, and **(E)** viability (z-axis) with other factors held at mid-levels. The color scale represents low (dark blue) to high (yellow) predicted response. All panels represent significant interaction effects (p ≤ 0.05) between the two factors shown on the x- and y-axes according to the interaction term p values from the ANOVA table. No significant interaction effects were identified for fibronectin secretion.

Normalized α-SMA fluorescence intensity (relative to the control) ranged from 0.16 to 1.81 across the tested ECM coating densities and soluble factor concentrations (Fig. 2A). Collagen I, laminin, and bFGF had significant effects on α-SMA expression (Table 1). Specifically, bFGF produced a negative linear effect on α-SMA, while collagen I demonstrated a positive quadratic effect, and laminin a negative quadratic effect (Fig. 2A). To test whether combinations of input variables produced effects on α-SMA intensity beyond those of single factors, we next examined interaction terms and response surface plots across the DOE space. All three interaction terms that reached significance for this response involved collagen III (Table 1). Specifically, collagen III exhibited significant interactions with laminin, FGF and vitamin D (Fig. 3A–C). Although collagen III did not independently drive α-SMA expression, these results suggest that collagen III plays an important modulatory role in fibroblast activation by shaping the influence of both ECM and soluble factors.

For fibronectin secretion, vitamin D emerged as the only significant input factor, with a pronounced positive quadratic effect, leading to decreased fibronectin secretion at low to mid (3–4 μM) vitamin D concentrations (Fig. 2B). Neither bFGF nor any ECM proteins showed significant main effects or interactions that impacted this response (Table 1). Furthermore, the absence of significant interaction terms between vitamin D and other input factors (Table 1) indicates that this soluble factor influences fibronectin secretion independently of the ECM substrates and bFGF.

Cell proliferation was significantly affected by vitamin D and bFGF (Table 1), with proliferation ranging from 1% to 25% of the total cell population. bFGF concentration demonstrated a positive linear relationship with proliferation, while vitamin D exhibited a negative quadratic effect, with maximal proliferation at a dosage between low and intermediate levels (1.8–3.8 μM; Fig. 2C). bFGF exhibited a robust main effect but did not have significant interaction terms with any of the ECM coatings or vitamin D (Table 1). On the other hand, vitamin D had a significant interaction with collagen III, with wider proliferation ranges observed in the response to vitamin D at high versus low collagen III coating densities (Fig. 3D).

Finally, cell viability displayed a range from 78.4% to 99.3% and was significantly influenced by both collagen I and vitamin D (Table 1). The factor effect plot revealed a slight negative quadratic relationship for collagen I (with maximal viability at 20–24 μg/cm^2^) and a more pronounced quadratic relationship for vitamin D, with a peak between 2–4 μM, closer to the lower end of the concentration range (Fig. 2D). Collagen I did not have significant interaction terms with any of the other input factors (Table 1). In contrast, vitamin D had a significant interaction with bFGF, with high doses of bFGF mitigating the cytotoxic effects of high vitamin D concentrations (Fig. 3E).

Overall, this DOE model demonstrates that combinations of ECM proteins and soluble factors can effectively modulate fibroblast phenotypes and suppress myofibroblastic markers. Whereas our single-factor preliminary experiments showed only partial effects, the DOE framework revealed that these inputs can act synergistically to produce a more quiescent phenotype.

### Validation of DOE Model Predictions

Next, we set out to validate the predictions of the DOE model experimentally. The desirability function algorithm was used to determine the optimal culture conditions to induce fibroblast quiescence by simultaneously minimizing α-SMA expression, fibronectin secretion, and proliferation, while maximizing viability (Opt.all; Table 2). These target outcomes were selected based on well-established phenotypes of quiescent fibroblasts: reduced expression of myofibroblastic markers, low ECM production, and low proliferation rates^4,42,47,48^. However, when inspecting the data collected to build the DOE model, we found that the conditions with the highest α-SMA intensity had low proliferation rates and vice-versa (Fig S1) Additionally, the literature specific to colonic fibroblasts includes conflicting reports on the relationship between proliferation and activation^50–52^. Thus, we also validated conditions predicted to minimize α-SMA expression, minimize fibronectin secretion, and maximize viability while excluding proliferation (Opt.all (-prolif); Table 2). Finally, we identified the culture conditions predicted to lead to the “best” (maximize) and “worst” (minimize) outcomes for each response variable (Table 2).

**Figure 4:**
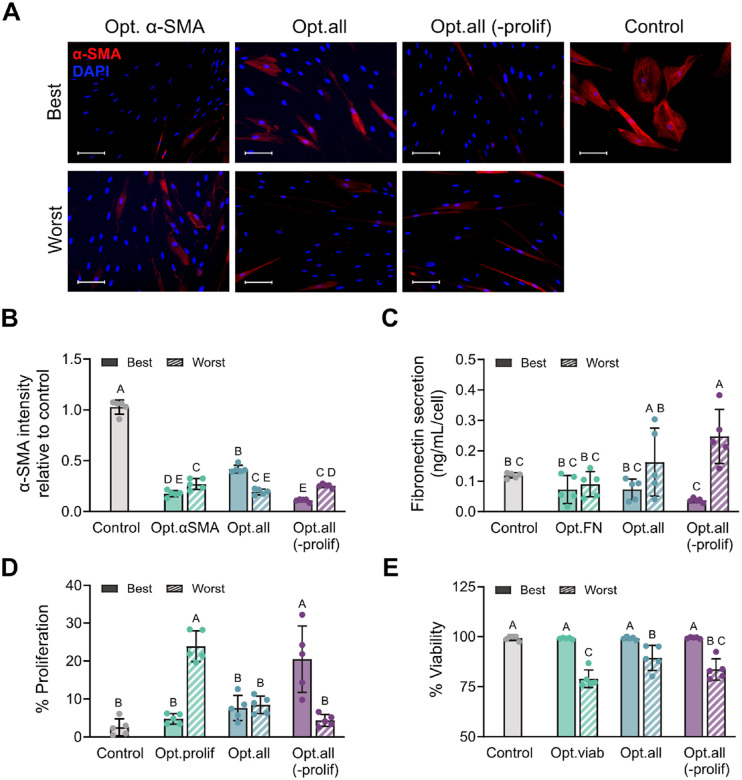
Experimental validation of the experimental conditions predicted to maximize or minimize each output response. The desirability function algorithm was applied to predict the optimal culture conditions for minimizing **(A-B)** α-SMA intensity, **(C)** fibronectin secretion, and **(D)** proliferation, and for maximizing **(E)** cell viability. Each optimization was tested in both its most desirable (best) and least desirable (worst) predicted form. Opt.α-SMA, Opt.FN, Opt.Prolif, and Opt.Viab refer to the conditions predicted when each response was individually optimized. Opt. All refers to the simultaneous optimization of all responses and Opt.All (–prolif) refers to the simultaneous optimization of all responses excluding proliferation. Untreated cells cultured on TCPS served as the control. Data are presented as mean ± SD (n = 5). Scale bar indicates 100 μm. Two-way ANOVA followed by Tukey’s multiple comparisons test. Different letters above bars indicate statistically significant differences between groups (p < 0.05); matching letters denote no significant difference (p > 0.05).

Experimental validation after 5 days of treatment revealed that all condition predicted by the DOE model led to statistically significant reductions in α-SMA intensity compared to the control - cells seeded on TCPS in media without supplements (Fig. 4A). α-SMA intensity was reduced 2- to 10-fold across conditions, even in the worst predictions, with the best “Opt.all (-prolif)” condition leading to the lowest expression levels of this myofibroblastic marker (Fig. 4B). Furthermore, the optimization that did include proliferation exhibited unexpected results, where the best prediction led to the highest α-SMA intensity levels compared both to the worst prediction and all other optimized conditions evaluated.

There were no statistically significant differences in fibronectin secretion between the best and worst predictions when all responses where simultaneously optimized (Fig. 4C). In contrast, there was a 15-fold difference in fibronectin secretion between the best and worst predictions for the optimization that excluded proliferation. The decrease in fibronectin production between all best predictions and the untreated control was not statistically significant. Similarly, there was no significant difference between the best and worst predictions when this response was optimized individually, likely due to high cell death rates in the worst Opt.FN condition (Fig S2).

Like fibronectin secretion, no significant differences in proliferation were observed between the best and worst predictions when all responses were optimized (Fig. 4D). When proliferation was excluded from the optimization, the rates for the best (20%) and worst (4%) predictions were the opposite of our initial assumptions about the quiescent phenotype in CCD-18Co fibroblasts. Additionally, the best prediction for those culture conditions was statistically similar to the rate observed when proliferation was maximized alone. These results suggest that α-SMA expression and proliferation rate may have an inverse relationship in colonic fibroblasts.

Cell viability remained above 99% for all of the “best” predictions compared to the control, indicating that all predicted culture conditions were highly effective in preventing cytotoxicity (Fig. 4E). As expected, cell viability dropped significantly for all “worst” predictions (78–89%), with the lowest viability observed for the worst prediction when this response was optimized alone. Collectively, these results demonstrate that the DOE model could accurately predict culture conditions optimized to achieve the desired cellular responses, with the optimization that excluded proliferation emerging as the best conditions to support quiescence.

### The Optimized Protocol Successfully Promotes Fibroblast Quiescence

Based on the validation of the DOE predictions (Fig. 4), we decided to proceed with the culture conditions for the “best” optimization when proliferation was excluded. The final optimized protocol consisted of culture on a coating composed of 27.3 μg/cm^2^ collagen I, 2 μg/cm^2^ collagen III, and 30 μg/cm^2^ laminin, and grown in media supplemented with 5.3 μM vitamin D and 20 ng/mL FGF. After growing the cells in these conditions for 5 days, we conducted an additional comprehensive analysis targeting key quiescence- and activation-related readouts. The control for these experiments consisted of conventional culture of fibroblasts on TCPS without media supplements. Cells treated with TGF-β_1_ (1 ng/mL) were also included as a positive control.

**Figure 5:**
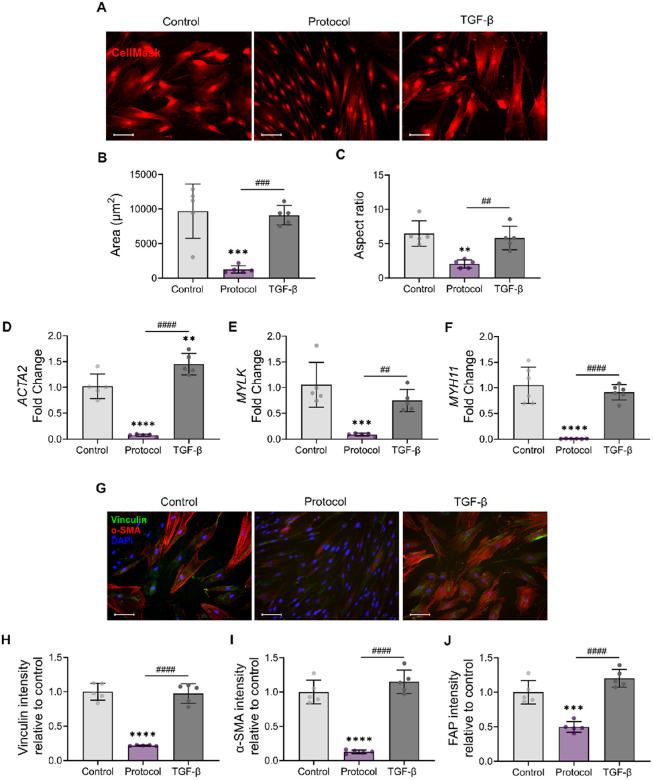
Analysis of cell morphology, and myofibroblastic marker expression in quiescent fibroblasts generated with the optimized protocol. **(A)** Immunofluorescence staining of the cell cytoplasm using Cell Mask Red (red). **(B-C)** Quantification of cell area and aspect ratio. (D-F)gene expression analysis of **(D)**ACTA2, **(E)** MYLK, **(F)** MYH11 via qRT-PCR, **(G)**Immunofluorescence staining of vinculin (green) and α-SMA (red), with nuclei counterstained with DAPI (blue). **(H-J)**Quantification of the staining intensity of the myofibroblastic protein markers **(H)** vinculin, **(I)** α-SMA and **(J)** FAP after 5 days of treatment. Data are presented as mean ± SD (n = 5). Scale bar indicates 100 μm. One-way ANOVA followed by Tukey’s multiple comparisons test. **p<0.005,*** p<0.0001 and ****p<0.0001 compared to the control and ##p<0.005, ## p<0.0001, ####p<0.0001 for comparisons shown.

Because quiescent fibroblasts are characterized by a reduced cell area and spindle-like shape^53,54^, we first assessed cell morphology. Staining of the cytoplasm resulted in the visualization of clear differences in cell shape between cells cultured following the optimized protocol and the control (Fig. 5A). Cells derived from the new protocol exhibited a thinner, spindled-shape morphology with long extensions, while cells in the control and TGF-β_1_ groups demonstrated a more spread, stellate-like morphology. Quantification of cell area and aspect ratio confirmed these observations. Cells cultured with the protocol adopted a more spindle-shaped, resting fibroblast phenotype, with a 9-fold and 2–3-fold decrease in cell area and aspect ratio, respectively, compared to both the control and the cells under TGF-β_1_ treatment (Fig. 5B–C).

Next, we assessed the expression of multiple activation-associated fibroblast genes^46,55,56^ —*ACTA2*, *MYH11*, *MYLK*, *AOC3*, and *NKX2–3* — via qRT-PCR. We observed a significant downregulation of *ACTA2* (Fig. 5D), *MYLK* (Fig. 5E), and *MYH11* (Fig. 5F), ranging from 8-fold to 10-fold, in the cells treated with the optimized conditions compared to the untreated control. In contrast, the TGF-β_1_–treated group showed either no change or significant upregulation of these genes compared to the control (Fig. 5D–F). The protocol also resulted in reduced expression of two additional colonic fibroblast activation markers, *AOC3* and *NKX2–3*, compared to the control (Fig S3A-B). Finally, we evaluated the expression of *SHOX2*, a marker that is found elevated in both quiescent and activated fibroblasts, but downregulated in myofibroblasts^46,58^. *SHOX2* expression was upregulated in both the control and protocol conditions compared to cells treated with TGF-β_1_ (Fig. S3C).

To supplement the DOE validation, we also quantified the protein expression levels of two myofibroblastic markers beyond α-SMA: the focal adhesion protein vinculin, and fibroblast activation protein (FAP)^52,59^. The optimized protocol led to a 4-fold reduction in the fluorescence intensities of vinculin and α-SMA staining compared to both the control and TGF-β_1_–treated groups (Fig. 5G–J). Similarly, FAP expression in fibroblasts treated with the optimized conditions was reduced by approximately 50% relative to the other experimental groups (Fig. 5J; Fig S3D). These data confirm that the optimized protocol successfully downregulates the expression of myofibroblastic markers at both the gene and protein levels.

**Figure 6:**
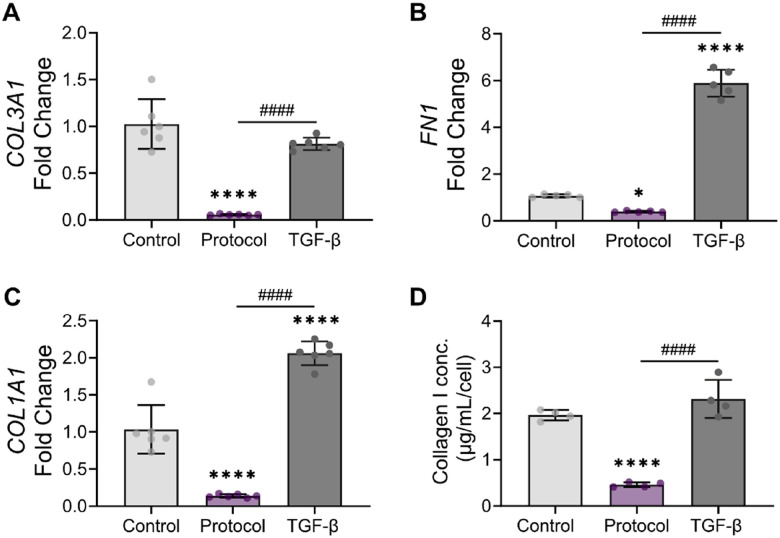
Quantification of ECM production by fibroblasts treated with the optimized conditions. **(A-C)** Gene expression analysis of **(A)** COL3A1, **(B)** FN1 and **(C)** COL1A1 after 5 days of treatment via qRT-PCR. **(D)** Quantification of secreted collagen I concentrations using ELISA. N = 4–6 replicates per condition. Data are presented as mean ± SD. Two-way ANOVA followed by Tukey’s multiple comparisons test. *p<0.05, ****p<0.0001 compared to the control, ####p<0.0001 compared to TGF-β_1_.

Because activated fibroblasts also produce more ECM^4,5^, we also confirmed that the optimized protocol decreased the production of ECM proteins beyond fibronectin. The cells treated with optimized cultured condition showed reduced transcription of the ECM genes *COL3A1* (Fig. 6A), *FN1* (Fig. 6B), and *COL1A1* (Fig. 6C) compared to cells in conventional culture. In contrast, treatment with TGF-β_1_ led to the significant upregulation of *COL1A1* and *FN* compared to the control. To confirm these changes at the protein level, we quantified secreted collagen type I in the supernatant (Fig. 6D). Both the control and TGF-β_1_–treated cells secreted relatively high amounts of collagen. In contrast, treatment with the optimized conditions led to a statistically significant 9-fold reduction in collagen I secretion.

Altogether, these results suggest that the protocol successfully induced a quiescent state in CCD-18Co colonic fibroblasts, as indicated by changes in cell morphology consistent with quiescence, and the downregulation of both myofibroblastic marker expression and ECM production.

## DISCUSSION

The spontaneous activation of fibroblasts upon *in vitro* culture on TCPS represents a significant limitation for experimental studies of intestinal fibrosis. In this study, we sought to identify key microenvironmental cues, ECM substrates and soluble factors, that modulate colonic fibroblast phenotype. Using DOE to systematically optimize culture conditions, we developed a protocol for inducing fibroblast quiescence in *in vitro* culture. Fibroblasts cultured using the quiescence protocol exhibited marked reductions in the expression of key myofibroblastic activation markers and decreased ECM secretion, while maintaining high cell viability. These results underscore the significant roles that ECM substrates and soluble agents play in shaping fibroblast phenotype, while also illustrating how DOE can uncover complex, non-linear relationships between these cues.

Vitamin D emerged as the most important input factor in the DOE model, with significant influence on all response variables through both direct effects or through interactions with other factors. This finding aligns with clinical and experimental reports on the role of vitamin D in intestinal fibrosis. Clinically, vitamin D deficiency is more prevalent in patients with Crohn’s disease than in the general population^60,61^. Furthermore, vitamin D receptor (VDR) expression is decreased in stenotic colonic tissues, and knocking down VDR has been shown to induce myofibroblastic activation and increased fibronectin deposition in colonic fibroblasts *in vitro*^29,62^.

These observations suggest that vitamin D may be critical for maintaining fibroblast quiescence. In fact, vitamin D supplementation inhibits myofibroblast activation in vitamin D-deficient mice with chronic colitis^28^, and reduces disease activity and relapse risk in Crohn’s patients^63–66^. Interestingly, we found that the effect of vitamin D on fibroblast phenotype is highly context dependent. For example, vitamin D alone did not significantly affect α-SMA expression; instead, its impact on this marker emerged through synergistic interactions with collagen III. Similarly, the influence of vitamin D on ECM secretion was complex: low doses suppressed fibronectin secretion, whereas high doses stimulated its production and induced cytotoxicity. Reflecting these conflicting findings, our final protocol includes vitamin D at an intermediate concentration of 5.3 μM. Overall, our findings confirm the important role of vitamin D in modulating colonic fibroblast activation, while also indicating that its antifibrotic effects depend on dosing and synergies with other microenvironmental cues.

Our findings also highlight bFGF as a potent regulator of fibroblast behavior, exerting significant effects on α-SMA intensity, cell proliferation, and viability. Not much is known about the specific contributions of bFGF to intestinal fibroblast activation. Available evidence suggests that bFGF supports intestinal wound healing^31,67,68^, with one study reporting increased fibroblast activation and ECM deposition after bFGF treatment in a mouse model of colonic anastomoses^68^. However, bFGF has been shown to suppress myofibroblast activation and TGF-β_1_ signaling in other fibroblast types^69–71^. Additional reports indicate that bFGF promotes regeneration in other tissues by increasing fibroblast proliferation, survival, and migration while impeding the full transition to a myofibroblastic phenotype^72–74^. In our optimized protocol, high concentrations of bFGF were associated with low α-SMA expression and high cell proliferation. Furthermore, bFGF counteracted the negative effects of vitamin D on cell viability. These data indicate bFGF might also induce a proliferative but non-contractile phenotype in intestinal fibroblasts.

ECM composition also emerged as a critical modulator of fibroblast behavior, with both collagen I and III having significant impacts on α-SMA expression. Collagens are the most abundant proteins in submucosal ECM, providing important structural support and maintaining stromal homeostasis in intestinal tissue; yet, their abnormal accumulation is a hallmark of fibrosis in Crohn’s disease^75–77^. The DOE model revealed a positive quadratic effect of collagen I on α-SMA intensity, with both low and high concentrations elevating its expression. While collagen III alone did not significantly affect α-SMA expression, it did exhibit significant interactions with other input factors that modulated this activation marker. The optimized coating composition for quiescence included high (27.3 μg/cm^2^) collagen I and low (5.3 μg/cm^2^) collagen III densities. Similar collagen I coating densities have been previously demonstrated to decrease the expression of myofibroblastic markers in valvular interstitial cells^24,41^. In contrast, fibroblasts isolated from strictures of Crohn’s disease patients deposit more collagen III compared to healthy controls^78^. Nonetheless, it is important to note that collagen coatings lack a fibrillar structure, which may impact the cellular response to these substrates due to differences in integrin signaling or the ability to generate tension compared to fibrillar configurations^79,80^. For example, hybrid nanofibril matrices composed of both collagen I and III at a 2:1 ratio lead to vaginal fibroblast activation more effectively than matrices composed of either protein alone^81^.

While the precise contribution of laminin to intestinal fibrosis remains poorly defined, evidence from other organs indicates that it functions not only as a structural epithelial scaffold but also as a dynamic regulator of fibrotic processes^43,82^, with effects that vary by tissue type and disease state. For example, reduced laminin expression in skeletal muscle and lung epithelium has been associated with increased cell death, fibrosis, and impaired tissue repair^83–85^, whereas excessive laminin accumulation is a feature of fibrotic liver tissue^82^. In our study, laminin exhibited a negative quadratic effect on α-SMA expression, with both high and low concentrations resulting suppressing this activation marker. Moreover, laminin displayed synergistic interactions with collagen III, further highlighting its context-dependent influence on fibroblast behavior.

One of the central findings of this study was that myofibroblastic marker expression and proliferation exhibited an inverse relationship in CCD-18Co fibroblasts. When building the DOE model, we initially sought to optimize quiescence by decreasing α-SMA expression, ECM production, and proliferation, based on published descriptions fibroblast activation in intestinal and other tissues^1,2,8,24,86,87^. The prediction validation process, however, revealed a different pattern for these colonic fibroblasts: high α-SMA expression was associated with low proliferation, whereas conditions that decreased α-SMA expression increased proliferation. Examination of the single-factor effects in the DOE model reinforced this dissociation, as high FGF and low vitamin D concentrations both reduced α-SMA while enhancing proliferation. In support of these observations, Simmons *et al*. previously demonstrated that treatment with TGF-β_1_ inhibits proliferation in CCD-18Co fibroblasts while increasing α-SMA expression and collagen production^88^. Similar phenomena have been described in other systems. TGF-β_1_ also stimulates corneal fibroblast activation without impacting proliferation^73^, and in cardiac fibroblasts, overexpression of α-SMA suppresses proliferation^42^. Collectively, these results indicate that myofibroblastic activation and proliferative capacity are not inherently linked but can shift independently depending on the tissue and stimulus.

Our preliminary experiments demonstrated that no single cue was sufficient to induce fibroblast quiescence, underscoring the need for a combinatorial approach. By applying DOE, we identified input combinations that produced robust quiescence while saving considerable time and resources compared to traditional trial-and-error methods^37^. This strategy not only revealed synergistic relationships between ECM proteins and soluble factors that would have been difficult to predict *a priori* but also highlighted the complexity of the intestinal microenvironment. More broadly, the development of a novel protocol to culture quiescent fibroblasts represents an important step toward the dissection of the mechanisms involved in intestinal fibrosis. We have previously demonstrated that quiescent valve fibroblasts are more sensitive to profibrotic stimuli like TGF-β_1_ and gut-derived metabolites than activated cells^24,89^. Thus, the establishment of a reliable method to sustain this phenotype *in vitro* opens the door to more precise investigation of the cellular and molecular cues that drive fibroblast activation. Looking ahead, these quiescent fibroblasts can serve as a foundation for designing more complex and physiologically relevant models that incorporate fibrotic stimuli, such as inflammatory cytokines or microbial metabolites, as well as 3D culture and co-culture approaches.

There are several limitations to this study that should be considered when interpreting the results. First, we developed this protocol using the CCD-18Co colonic fibroblast cell line. While this cell line is widely used and experimentally tractable, it does not fully capture the heterogeneity of primary intestinal fibroblasts. Future validation of the protocol with primary cells derived from human intestinal tissue will be essential to confirm and extend our findings. Second, although the DOE approach enabled systematic exploration of selected ECM substrates and soluble factors, we did not include other stimuli that influence intestinal fibroblast activation, such as inflammatory cytokines or mechanical forces. Even so, we were successful in inducing fibroblast quiescence using a limited set of inputs, showing that relatively simple culture modifications can have a strong impact on cell state. This simplicity also makes the protocol easier for other researchers to reproduce in future studies at both small and large scales. Finally, the culture duration in this study was relatively short – 5 days. Our prior work with valvular interstitial cells showed that longer culture periods can further stabilize the quiescent phenotype^24^, raising the possibility that extended time courses could enhance the effects observed here.

In conclusion, our study presents a robust protocol to effectively induce quiescence in CCD-18Co colonic fibroblasts by leveraging statistical optimization through DOE. This method led to significantly reduced activation markers, including the downregulation of several myofibroblastic proteins and genes, resulting in a resting spindle-shaped fibroblast phenotype. By enabling the simultaneous optimization of multiple cellular behaviors, the DOE approach offers a powerful strategy for defining culture conditions that better recapitulate physiologic fibroblast states. Furthermore, our findings highlight the critical role of ECM coatings and soluble factors as modulators of fibroblast activation and demonstrate the importance of microenvironmental cues in regulating cellular behavior. By generating a more physiologically relevant model of healthy fibroblasts, we provide a valuable *in vitro* platform for researchers seeking to study the triggers and underlying mechanisms associated with colonic fibrosis.

## MATERIALS AND METHODS

### ECM protein coatings

Tissue culture-treated well plates were coated with collagen type I (Sigma Aldrich), collagen type III (Advanced Biomatrix) or laminin (Gibco) in bicarbonate buffer (pH 9.6) at one of three densities (2, 15, or 30 μg/cm^2^). The plates were incubated overnight at 4°C, then washed with sterile PBS prior to cell culture to remove residual coating solution.

### Fibroblast culture for single factor experiments

CCD-18Co colonic fibroblasts (ATCC CRL-1459) were expanded in TCPS flasks using Eagle’s Minimum Essential Medium (EMEM; Fisher Scientific) supplemented with 10% fetal bovine serum (FBS; Sigma Aldrich) in a humidified incubator at 37°C with 5% CO_2_. Upon reaching 80% confluency, the cells were trypsinized, centrifuged at 300 RCF for 5 minutes, and seeded at a density of 5,000 cells/cm^2^ in EMEM containing 2% FBS.

For the initial exploratory experiments, only one factor was varied at a time to assess its individual effect on fibroblast phenotype. To evaluate the impact of individual ECM protein coatings, cells were seeded onto plates coated with either collagen I, collagen III, or laminin at three densities (2, 15, or 30 μg/cm^2^). In the soluble factor experiments, cells were seeded on non-coated plates; following a 24-hour incubation period, the media was replaced with EMEM supplemented with 2% FBS and either human basic FGF (1, 10, and 20 ng/mL; ThermoFisher Scientific), or vitamin D (1, 5, and 10 μM; Fisher Scientific). For all experiments, the culture media was refreshed every other day. Control wells consisted of cells seeded on non-coated TCPS and maintained in 2% FBS without additional supplements. On day 5, cell viability was assessed, and the cells were fixed for downstream analyses, including measurement of α-SMA intensity and proliferation.

### Quantification of proliferation

Proliferation was assessed using a Click-iT EdU Alexa Fluor 488 Imaging Kit (Invitrogen) according to the manufacturer’s instructions. Briefly, on day 4 of culture, the cells were pulsed with 10 μM EdU for 18 hours to label newly synthesized DNA. The cells were then fixed with 10% formalin for 15 minutes at room temperature, permeabilized with 0.5% Triton X-100 for 20 minutes and incubated with the Click-iT reaction cocktail for 30 minutes protected from light at room temperature. Cell nuclei were counterstained with 4’,6-diamidino-2-phenylindole (DAPI) (Thermo Fisher Scientific, 1:1000 in PBS). Fluorescent images were acquired using a Keyence fluorescence microscope (BZ-X800 Series). The percentage of proliferating cells was determined by quantifying the number of EdU-positive nuclei (green fluorescence) relative to the total number of DAPI-stained nuclei (blue fluorescence) using Keyence analyzer software. The proliferation rate was calculated as:

$$\text{Proli}\;\text{feration}(\%)=\frac{\text{Number}\;\text{of}\;{\text{EdU}}^{\text{+}}\text{cells}}{\text{Total}\;\text{number}\;\text{of}\;\text{cells}}\times 100$$

### Immunocytochemistry for myofibroblastic markers

α-Smooth muscle actin (α-SMA) expression was evaluated using standard immunocytochemistry techniques. Briefly, cells were fixed with 10% formalin for 15 minutes, permeabilized with 0.1% Triton X-100 for 20 minutes and blocked with 3% bovine serum albumin (BSA) in PBS for 30 minutes. Cells were incubated for 2 hours with the following primary antibodies diluted in 1% BSA: mouse monoclonal anti–α-SMA (clone 1A4, Sigma Aldrich; 1:500), recombinant rabbit monoclonal anti–FAP (clone 5H0Y9, Invitrogen; 1:100), and recombinant rabbit monoclonal anti–Vinculin (clone 42H89L44, Invitrogen; 1:100). After three washes with PBS to remove unbound antibodies, the cells were incubated with a goat anti-mouse IgG secondary antibody conjugated to Alexa Fluor 594 (Invitrogen; 1:1000 dilution in 1% BSA) and goat anti-rabbit IgG secondary antibody conjugated to Alexa Fluor 488 (Invitrogen; 1:1000 dilution in 1% BSA) for 1 hour, followed by another wash step with PBS. Cell nuclei were counterstained with DAPI (1:1000 in PBS) for 5 minutes and washed with PBS to prepare for visualization. All staining steps were conducted at room temperature.

Immunofluorescence images were captured using a Keyence fluorescence microscope (BZ-X800 series), acquiring images in separate channels for DAPI (blue, nuclear stain) and the desired marker channel (red or green) for each field of view. For every well, six representative fields were selected and imaged in batch mode, recording the blue, red or green, and corresponding overlay images for each location. Image sets from each condition (≥ 24 images per condition) were then analyzed using CellProfiler Image Analysis (version 4.2.5) software^90^. Nuclei were identified as primary objects in the DAPI channel using adaptive thresholding with the Otsu method, and cytoplasmic regions were segmented as secondary objects in the marker channel using the propagation method, applying adaptive thresholding with the Otsu method along with a threshold correction factor of 1 and an adaptive window size of 100. For each cell, mean cytoplasmic marker intensity was calculated by dividing the summed pixel intensity within the secondary object by the total number of pixels defining the cytoplasmic region. Identical image processing parameters were applied across experimental conditions. The average cell fluorescence intensity for each condition was normalized to the average intensity in the control on the same well plate.

### Assessment of cell viability

Cell viability was evaluated on day 5 using a Live/Dead viability assay kit (Invitrogen) following the manufacturer’s protocol. After removing all existing culture media, cells were incubated with a 1:2 dilution of Live/Dead solution in fresh culture media (prepared by mixing equal volumes of the assay reagents and fresh media) for 15 minutes at room temperature in the dark. Post-incubation, the cells were imaged using a Keyence BZ-X800 fluorescence microscope with live cells fluorescing green and dead cells fluorescing red. A constant number of field of views were imaged for each well and the number of live/dead cell count per well was quantified using the Keyence BZ-X800 image analyzer software. The viability percentage was then calculated as the ratio of live cells to the total cell number, multiplied by 100%.

### Quantification of ECM production

Fibronectin and collagen secretion were quantified using commercially available Human Fibronectin ELISA Kit (RayBiotech) and Human Pro-Collagen I alpha 1 DuoSet ELISA (DY6220–05, R&D Systems) following the manufacturer’s instructions. At the end of the 5-day culture period, cell culture media supernatant was collected from the cells for analysis. For the collagen ELISA, an additional control was included consisting of media collected from ECM-coated wells in the absence of cells to account for collagen present in the coatings that might interfere with the readings. The samples were diluted 1:10 and 1:20 in assay diluent for COL1A1 and FN, respectively, and added to plates pre-coated with capture antibody, with each condition tested in six replicates. The samples and standards were incubated for two and a half hours. Following a washing step, biotin-labeled detection antibody was added for a one-hour incubation. After washing to remove unbound biotinylated antibodies, HRP-conjugated streptavidin was incubated for 30 minutes. Finally, the chromogenic substrate, 1-Step Ultra TMB-ELISA Substrate Solution (Fisher Scientific), was added and incubated for 30 minutes. The reaction was stopped with sulfuric acid, and the absorbance was measured at 450 nm using an absorbance plate reader (Molecular devices SpectraMax^®^ iD3). All steps of this ELISA protocol were performed at room temperature. The fibronectin concentration in the media was calculated using the standard curve and normalized to the cell count for the corresponding well.

### Design of Experiments (DOE)

DOE statistical optimization was employed to assess the effects and interactions of key ECM components and soluble factors on cellular phenotypes. A Box-Behnken design was generated using Stat-Ease 360 software (Stat-Ease Inc., version 23.1.6) to systematically evaluate five continuous input variables. The input variables include 3 ECM coatings (collagen I, collagen III, and laminin) at seeding densities ranging from 2–30 μg/cm^2^, and 2 soluble factors: vitamin D (1–10 μM), and bFGF (1–20 ng/mL), Each factor was examined at three equidistant levels (low point, midpoint, high point), resulting in a total of 42 unique experimental conditions (Supplementary Table 1), including two centrally repeated runs to assess system variance and optimize the statistical power of the design.

For each experimental condition, well plates were coated with the appropriate ECM protein concentrations as previously described. CCD-18Co colonic fibroblasts were seeded at a density of 5,000 cells/cm^2^ in EMEM containing 2% FBS. After 24 hours, the media was replaced with 2% serum EMEM supplemented with bFGF and vitamin D according to the assigned condition. The culture media was refreshed every other day throughout the experiment for a total of 5 days of treatment.

All extraneous variables, such as cell line batch, passage number, and reagents, were meticulously controlled to ensure that the observed effects could be attributed to the factors under investigation. Each well plate included internal control wells for baseline comparison and normalization. The primary response variables measured were α-SMA intensity, fibronectin secretion, and cell viability. Multiple linear regression models were constructed for each response as a function of the input variables, with statistical significance defined as p < 0.05. Model performance was evaluated using coefficient of determination (R^2^) to describe the proportion of variance explained by the model.

### Validation of the DOE model

Building on the DOE model, we used Stat-Ease 360 software to predict optimal combinations of the five input variables that would: (1) simultaneously minimize α-SMA intensity, fibronectin secretion and proliferation while maximizing cell viability, (2) optimize all responses as described while excluding proliferation, or (3) individually optimize each of these outcomes, each in their best desirable and worst desirable predictions (Table 2). Each predicted combination was subsequently validated experimentally using the same protocols described above, ensuring consistency across all tests.

### Cell culture conditions for validation of the optimized quiescence protocol

Culture wells were coated with 27.3 μg/cm^2^ collagen I, 2 μg/cm^2^ collagen III, and 30 μg/cm^2^ laminin according to the optimized protocol excluding proliferation (Table 2). CCD-18Co colonic fibroblasts were seeded on these wells at 5,000 cells/cm^2^ in EMEM supplemented with 2% FBS. After plating, the cells were allowed to attach for 24 hours, and the medium was replaced with EMEM + 2% FBS containing 5.3 μM vitamin D and 20 ng/mL FGF. The media was refreshed every other day, and the treatments were maintained for 5 days. The negative control group was cultured under identical conditions without ECM coatings or vitamin D/FGF supplementation. Treatment with TGF-β1 (1 ng/mL) was used as a positive control.

### Analysis of gene expression via qRT-PCR

Gene expression levels were assessed via quantitative reverse transcription-polymerase chain reaction (qRT-PCR) after growing the cells for 5 days with the optimized quiescence protocol. Total RNA was extracted using the RNeasy Mini Kit (Qiagen), following the manufacturer’s instructions. The isolated RNA was then reverse transcribed into cDNA with the High-Capacity cDNA Reverse Transcription Kit (Applied Biosystems). qRT-PCR was subsequently conducted using TaqMan Gene Expression Assays (Applied Biosystems) targeting fibroblast activation markers—including α-SMA (*ACTA2*), Myosin Heavy Chain 11 (*MYH11*), Myosin Light Chain Kinase (*MYLK*), Amine Oxidase Copper Containing 3 (*AOC3*), Short Stature Homeobox 2 (*SHOX2*), and NK2 homeobox 3 (*NKX2–3*) as well as the extracellular matrix proteins collagen Type I (*COL1A1*) and collagen Type III (*COL3A1*) and fibronectin (FN1). Gene expression was quantified using the comparative Ct (ΔΔCt) method, normalized to the endogenous control (GAPDH), and expressed relative to the untreated control condition.

### Evaluation of cell morphology

To quantify cell morphology, fixed and permeabilized cells were stained with HCS Cell Mask Red (Invitrogen) according to the manufacturer’s instructions. A 10 mg/mL stock solution was prepared by dissolving 250 μg of stain in 25 μL of DMSO. The working (1x) staining solution was prepared from this stock and applied to the cells for 30 min at room temperature, protected from light. The wells were then washed three times with PBS, and cell nuclei were counterstained with DAPI (1:1000 in PBS) for 5 min, followed by additional PBS washes. Cytoplasmic and nuclear fluorescence was subsequently imaged using a Keyence BZ-X800 fluorescence microscope. Cell area and aspect ratio were automatically quantified in Fiji (ImageJ 1.54p). Images were converted to 8-bit and segmented using Otsu’s threshold and binarization. Measurement settings for Area and Fit Ellipse were enabled, and cells were analyzed via “Analyze Particles.” Batch macro processing and export facilitated quantification of 50 cells per condition across 4 replicates. Aspect ratio was calculated as the major axis divided by the minor axis for each cell.

### Statistical analysis

For all experiments outside of the DOE model, statistical analyses were performed using GraphPad Prism software. Data are presented as mean ± standard deviation. For each condition, experiments included 3–6 technical replicates. Differences between groups were assessed using one-way ANOVA followed by Tukey’s post hoc test for multiple comparisons, and *p* values less than 0.05 were considered statistically significant.

## Supplementary Files

This is a list of supplementary files associated with this preprint. Click to download.

- SupplementaryFiguresnpj.docx
- SupplementaryTable.xlsx
- SupplementaryCellProfiler.zip

## Figures and Tables

**Table 1:** ANOVA table for the DOE model, highlighting statistically significant relationships between input and output variables with their associated p values. R^2^: coefficient of correlation. Confidence level 95%. Non-significant values are stated with a dash (−).

Response	α-SMA intensity	Fibronectin production	Proliferation	Viability
*p* values				
Model	< 0.0001	0.0040	0.0002	< 0.0001
Col I	-	-	-	0.0030
Col III	-	-	-	-
Lam	-	-	-	-
Vit D	-	< 0.0001	< 0.0001	< 0.0001
FGF	< 0.0001	-	< 0.0001	-
Col I^2^	0.0071	-	-	0.0277
Col III^2^	-	-	-	-
Lam^2^	0.0051	-	-	-
Vit D^2^	-	0.0094	0.0024	< 0.0001
FGF^2^	-	-	-	-
Col I × Col III	-	-	-	-
Col I × Lam	-	-	-	-
Col I × Vit D	-	-	-	-
Col I × FGF	-	-	-	-
Col III × Lam	0.0211	-	-	-
Col III × Vit D	0.0006	-	0.0200	-
Col III × FGF	0.0455	-	-	-
Lam × Vit D	-	-	-	-
Lam × FGF	-	-	-	-
Vit D × FGF	-	-	-	0.0505
Lack of fit	0.6747	0.9067	0.8410	0.3979
Validation metrics				
Total sample size (*N*)	42	42	42	42
Degree of freedom (df)	20	20	20	20
*R* ^2^	0.79	0.57	0.52	0.91
Adjusted *R*^2^	0.72	0.52	0.50	0.90
Predicted *R*^2^	0.59	0.46	0.44	0.84

**Table 2:** Culture conditions predicted to maximize or minimize cell outcomes. Col I – collagen I, Col III – collagen III, Lam – laminin, Vit D – vitamin D.

Response		Col I (μg/cm^2^)	Col III (μg/cm^2^)	Lam (μg/cm^2^)	Vit D (μM)	bFGF (ng/mL)
Optimize all responses (minimize α-SMA intensity, minimize fibronectin secretion, maximize viability, minimize proliferation)	Best	16.3	27	2	1	1
	Worst	30	2	2	9.16	8.62
Optimize all responses without proliferation (minimize α-SMA intensity, minimize fibronectin secretion, maximize viability)	Best	27.3	2	30	5.3	20
	Worst	2	30	24.14	10	1.13
Optimize α-SMA intensity	Best	9.3	8.4	30	8.8	18.5
	Worst	30	2.85	5.3	1.1	2
Optimize Fibronectin secretion	Best	2	16	2	5.5	10.5
	Worst	25.35	5.8	7.3	9.7	6.5
Optimize Proliferation	Best	30	30	3	9.4	1.4
	Worst	3	6	5.3	3.6	18.8
Optimize Viability	Best	16.1	4.2	21	4	17.3
	Worst	2	29	16.6	10	1

## Data Availability

Data generated or analyzed during this study are included in this published article and its supplementary files.

## CODE AVAILABILITY

The CellProfiler pipelines used to analyze fluorescence intensity and cell morphology are available as a supplementary file.

## References

[1] Rieder F. & Fiocchi C. Intestinal fibrosis in IBD—a dynamic, multifactorial process. Nat Rev Gastroenterol Hepatol 6, 228–235 (2009). 19347014 10.1038/nrgastro.2009.31

[2] Latella G. & Rieder F. Intestinal fibrosis: ready to be reversed. Current Opinion in Gastroenterology 33, 239–245 (2017). 28402994 10.1097/MOG.0000000000000363 PMC5572460

[3] Lenti M. V. & Di Sabatino A. Intestinal fibrosis. Molecular Aspects of Medicine 65, 100–109 (2019). 30385174 10.1016/j.mam.2018.10.003

[4] Roulis M. & Flavell R. A. Fibroblasts and myofibroblasts of the intestinal lamina propria in physiology and disease. Differentiation 92, 116–131 (2016). 27165847 10.1016/j.diff.2016.05.002

[5] Chalkidi N., Paraskeva C. & Koliaraki V. Fibroblasts in intestinal homeostasis, damage, and repair. Front. Immunol. 13, 924866 (2022). 36032088 10.3389/fimmu.2022.924866 PMC9399414

[6] Alfredsson J. & Wick M. J. Mechanism of fibrosis and stricture formation in Crohn’s disease. Scand J Immunol 92, e12990 (2020). 33119150 10.1111/sji.12990 PMC7757243

[7] Stallmach A., Schuppan D., Riese H. H., Matthes H. & Riecken E. O. Increased collagen type III synthesis by fibroblasts isolated from strictures of patients with Crohn’s disease. Gastroenterology 102, 1920–1929 (1992). 1587410 10.1016/0016-5085(92)90314-o

[8] Johnson L. A. Matrix Stiffness Corresponding to Strictured Bowel Induces a Fibrogenic Response in Human Colonic Fibroblasts: Inflammatory Bowel Diseases 19, 891–903 (2013). 23502354 10.1097/MIB.0b013e3182813297 PMC3766259

[9] Soroosh A. Crohn’s Disease Fibroblasts Overproduce the Novel Protein KIAA1199 to Create Proinflammatory Hyaluronan Fragments. Cellular and Molecular Gastroenterology and Hepatology 2, 358–368.e4 (2016). 27981209 10.1016/j.jcmgh.2015.12.007 PMC5042354

[10] Chang J. T. Pathophysiology of Inflammatory Bowel Diseases. New England Journal of Medicine 383, 2652–2664 (2020). 33382932 10.1056/NEJMra2002697

[11] Pompeu B. F. Extended versus limited mesenteric excision in bowel resection for Crohn’s disease: a meta-analysis and systematic review. Tech Coloproctol 29, 80 (2025). 40057916 10.1007/s10151-024-03108-w PMC11891095

[12] Santacroce G., Lenti M. V. & Di Sabatino A. Therapeutic Targeting of Intestinal Fibrosis in Crohn’s Disease. Cells 11, 429 (2022). 35159238 10.3390/cells11030429 PMC8834168

[13] Tomasek J. J., Gabbiani G., Hinz B., Chaponnier C. & Brown R. A. Myofibroblasts and mechano-regulation of connective tissue remodelling. Nat Rev Mol Cell Biol 3, 349–363 (2002). 11988769 10.1038/nrm809

[14] Hinz B., Celetta G., Tomasek J. J., Gabbiani G. & Chaponnier C. Alpha-Smooth Muscle Actin Expression Upregulates Fibroblast Contractile Activity. MBoC 12, 2730–2741 (2001). 11553712 10.1091/mbc.12.9.2730 PMC59708

[15] Yeung T. Effects of substrate stiffness on cell morphology, cytoskeletal structure, and adhesion. Cell Motility 60, 24–34 (2005). 10.1002/cm.20041 15573414

[16] Jiang H. & Grinnell F. Cell–Matrix Entanglement and Mechanical Anchorage of Fibroblasts in Three-dimensional Collagen Matrices. MBoC 16, 5070–5076 (2005). 16107563 10.1091/mbc.E05-01-0007 PMC1266407

[17] Grinnell F., Ho C.-H., Tamariz E., Lee D. J. & Skuta G. Dendritic Fibroblasts in Three-dimensional Collagen Matrices. MBoC 14, 384–395 (2003). 12589041 10.1091/mbc.E02-08-0493 PMC149979

[18] Hinz B. The myofibroblast: Paradigm for a mechanically active cell. Journal of Biomechanics 43, 146–155 (2010). 19800625 10.1016/j.jbiomech.2009.09.020

[19] Arora P. D. & McCulloch C. A. G. Dependence of collagen remodelling on α-smooth muscle actin expression by fibroblasts. Journal of Cellular Physiology 159, 161–175 (1994). 8138584 10.1002/jcp.1041590120

[20] Stewart D. C. Quantitative assessment of intestinal stiffness and associations with fibrosis in human inflammatory bowel disease. PLoS One 13, e0200377 (2018). 29995938 10.1371/journal.pone.0200377 PMC6040714

[21] Denys H. Differential impact of TGF-β and EGF on fibroblast differentiation and invasion reciprocally promotes colon cancer cell invasion. Cancer Letters 266, 263–274 (2008). 18423981 10.1016/j.canlet.2008.02.068

[22] Laudadio I. ZNF281 Promotes Colon Fibroblast Activation in TGFβ1-Induced Gut Fibrosis. IJMS 23, 10261 (2022). 36142169 10.3390/ijms231810261 PMC9499662

[23] Johnson L. A. Novel Rho/MRTF/SRF Inhibitors Block Matrix-stiffness and TGF-β–Induced Fibrogenesis in Human Colonic Myofibroblasts: Inflammatory Bowel Diseases 20, 154–165 (2014). 24280883 10.1097/01.MIB.0000437615.98881.31 PMC4893808

[24] Porras A. M. Robust Generation of Quiescent Porcine Valvular Interstitial Cell Cultures. Journal of the American Heart Association 6, e005041 (2017). 28292746 10.1161/JAHA.116.005041 PMC5524027

[25] Couchman J. R., Höök M., Rees D. A. & Timpl R. Adhesion, growth, and matrix production by fibroblasts on laminin substrates. Journal of Cell Biology 96, 177–183 (1983). 6681817 10.1083/jcb.96.1.177 PMC2112271

[26] Jabaji Z. Type I Collagen as an Extracellular Matrix for the In Vitro Growth of Human Small Intestinal Epithelium. PLOS ONE 9, e107814 (2014). 25222024 10.1371/journal.pone.0107814 PMC4164635

[27] Fletcher J., Cooper S. C., Ghosh S. & Hewison M. The Role of Vitamin D in Inflammatory Bowel Disease: Mechanism to Management. Nutrients 11, 1019 (2019). 31067701 10.3390/nu11051019 PMC6566188

[28] Tao Q. Vitamin D Prevents the Intestinal Fibrosis Via Induction of Vitamin D Receptor and Inhibition of Transforming Growth Factor-Beta1/Smad3 Pathway. Dig Dis Sci 60, 868–875 (2015). 25326845 10.1007/s10620-014-3398-6

[29] Yu M. Vitamin D receptor inhibits EMT via regulation of the epithelial mitochondrial function in intestinal fibrosis. J Biol Chem 296, 100531 (2021). 33713706 10.1016/j.jbc.2021.100531 PMC8054199

[30] Danopoulos S., Schlieve C. R., Grikscheit T. C. & Al Alam D. Fibroblast Growth Factors in the Gastrointestinal Tract: Twists and Turns. Developmental Dynamics 246, 344–352 (2017). 28198118 10.1002/dvdy.24491

[31] Song X. Growth Factor FGF2 Cooperates with Interleukin-17 to Repair Intestinal Epithelial Damage. Immunity 43, 488–501 (2015). 26320657 10.1016/j.immuni.2015.06.024

[32] Yasui H. Role of Fibroblast Growth Factor-2 in the Expression of Matrix Metalloproteinases and Tissue Inhibitors of Metalloproteinases in Human Intestinal Myofibroblasts. Digestion 69, 34–44 (2004). 14755151 10.1159/000076545

[33] Murphy K. C. Multifactorial Experimental Design to Optimize the Anti-Inflammatory and Proangiogenic Potential of Mesenchymal Stem Cell Spheroids. Stem Cells 35, 1493–1504 (2017). 28276602 10.1002/stem.2606 PMC5446296

[34] Cadena I. A. Engineering high throughput screening platforms of cervical cancer. J Biomedical Materials Res 111, 747–764 (2023). 10.1002/jbm.a.37522 36861788

[35] Weissman S. A. & Anderson N. G. Design of Experiments (DoE) and Process Optimization. A Review of Recent Publications. Org. Process Res. Dev. 19, 1605–1633 (2015).

[36] Bruns R. E. Statistical Design–Chemometrics. (Elsevier, Boston, 2006).

[37] G. Harris C., Semprini L., E. Rochefort W. & C. Fogg K. Statistical optimization of cell–hydrogel interactions for green microbiology – a tutorial review. RSC Sustainability 2, 3750–3768 (2024). 39464839 10.1039/d4su00400k PMC11499971

[38] Wang Y. Intestinal Fibrosis in Inflammatory Bowel Disease and the Prospects of Mesenchymal Stem Cell Therapy. Frontiers in Immunology 13, (2022). 10.3389/fimmu.2022.835005 PMC8971815 35370998

[39] Le C. C. Functional Interplay Between Collagen Network and Cell Behavior Within Tumor Microenvironment in Colorectal Cancer. Front Oncol 10, 527 (2020). 32426274 10.3389/fonc.2020.00527 PMC7204546

[40] Cameron G. J., Alberts I. L., Laing J. H. & Wess T. J. Structure of Type I and Type III Heterotypic Collagen Fibrils: An X-Ray Diffraction Study. Journal of Structural Biology 137, 15–22 (2002). 12064929 10.1006/jsbi.2002.4459

[41] Rodriguez K. J. & Masters K. S. Regulation of valvular interstitial cell calcification by components of the extracellular matrix. Journal of Biomedical Materials Research Part A 90A, 1043–1053 (2009). 10.1002/jbm.a.32187 PMC4230299 18671262

[42] Shinde A. V., Humeres C. & Frangogiannis N. G. The role of α-smooth muscle actin in fibroblast-mediated matrix contraction and remodeling. Biochim Biophys Acta Mol Basis Dis 1863, 298–309 (2017). 27825850 10.1016/j.bbadis.2016.11.006 PMC5163362

[43] Liu P., Chen H., Yan L. & Sun Y. Laminin α5 modulates fibroblast proliferation in epidural fibrosis through the PI3K/AKT/mTOR signaling pathway. Molecular Medicine Reports 21, 1491–1500 (2020). 32016453 10.3892/mmr.2020.10967 PMC7003017

[44] Biggs R. M. & Bradshaw A. D. Organ fibrosis: beyond collagen I expression. Fibroblast phenotype and basement membrane proteins. American Journal of Physiology-Cell Physiology 328, C2023–C2031 (2025). 40353336 10.1152/ajpcell.00077.2025 PMC12168137

[45] Chen P.-Y. & Simons M. FGF-TGFβ dialogues, endothelial cell to mesenchymal transition, and atherosclerosis. Curr Opin Lipidol 29, 397–403 (2018). 30080704 10.1097/MOL.0000000000000542 PMC6290915

[46] Hsia L.-T. Myofibroblasts are distinguished from activated skin fibroblasts by the expression of AOC3 and other associated markers. Proc Natl Acad Sci U S A 113, E2162–2171 (2016). 27036009 10.1073/pnas.1603534113 PMC4839407

[47] Talele N. P., Fradette J., Davies J. E., Kapus A. & Hinz B. Expression of α-Smooth Muscle Actin Determines the Fate of Mesenchymal Stromal Cells. Stem Cell Reports 4, 1016–1030 (2015). 26028530 10.1016/j.stemcr.2015.05.004 PMC4471834

[48] Mitra M., Ho L. D. & Coller H. A. An In Vitro Model of Cellular Quiescence in Primary Human Dermal Fibroblasts. in Cellular Quiescence: Methods and Protocols (ed. Lacorazza H. D.) 27–47 (Springer, New York, NY, 2018). doi:10.1007/978-1-4939-7371-2_2. PMC5718883 29030810

[49] Marescal O. & Cheeseman I. M. Cellular Mechanisms and Regulation of Quiescence. Dev Cell 55, 259–271 (2020). 33171109 10.1016/j.devcel.2020.09.029 PMC7665062

[50] McKaig B. C., Hughes K., Tighe P. J. & Mahida Y. R. Differential expression of TGF-β isoforms by normal and inflammatory bowel disease intestinal myofibroblasts. American Journal of Physiology-Cell Physiology 282, C172–C182 (2002). 11742810 10.1152/ajpcell.00048.2001

[51] Johnson L. A. Novel Rho/MRTF/SRF Inhibitors Block Matrix-stiffness and TGF-β–Induced Fibrogenesis in Human Colonic Myofibroblasts: Inflammatory Bowel Diseases 20, 154–165 (2014). 24280883 10.1097/01.MIB.0000437615.98881.31 PMC4893808

[52] Johnson L. A. Matrix Stiffness Corresponding to Strictured Bowel Induces a Fibrogenic Response in Human Colonic Fibroblasts. Inflamm Bowel Dis 19, 891–903 (2013). 23502354 10.1097/MIB.0b013e3182813297 PMC3766259

[53] Papadopoulou A. Reacquisition of a spindle cell shape does not lead to the restoration of a youthful state in senescent human skin fibroblasts. Biogerontology 21, 695–708 (2020). 32533368 10.1007/s10522-020-09886-8

[54] Monika P. Human primary chronic wound derived fibroblasts demonstrate differential pattern in expression of fibroblast specific markers, cell cycle arrest and reduced proliferation. Experimental and Molecular Pathology 127, 104803 (2022). 35679887 10.1016/j.yexmp.2022.104803

[55] Song J. TL1A Promotes Fibrogenesis in Colonic Fibroblasts via the TGF-β1/Smad3 Signaling Pathway. CURR MED SCI 44, 519–528 (2024). 38842774 10.1007/s11596-024-2875-1

[56] Ahmad Zawawi S. S. Identification of AOC3 and LRRC17 as Colonic Fibroblast Activation Markers and Their Potential Roles in Colorectal Cancer Progression. Asian Pac J Cancer Prev 24, 3099–3107 (2023). 37774061 10.31557/APJCP.2023.24.9.3099 PMC10762737

[57] Fortunato A. & Luca B. An Analytical Method for the Quantification of hERG1 Channel Gene Expression in Human Colorectal Cancer. Diagn Mol Pathol 22, (2013). 10.1097/PDM.0b013e31828e55c7 24193004

[58] Musa M. Interactions between myofibroblasts and colorectal cancer (CRC) epithelial cell lines. (University of Oxford, 2018).

[59] Wikberg M. L. High intratumoral expression of fibroblast activation protein (FAP) in colon cancer is associated with poorer patient prognosis. Tumor Biol. 34, 1013–1020 (2013). 10.1007/s13277-012-0638-2 PMC3597266 23328994

[60] Fletcher J., Cooper S. C., Ghosh S. & Hewison M. The Role of Vitamin D in Inflammatory Bowel Disease: Mechanism to Management. Nutrients 11, 1019 (2019). 31067701 10.3390/nu11051019 PMC6566188

[61] Ham M. Vitamin D Levels in Adults with Crohn’s Disease Are Responsive to Disease Activity and Treatment. Inflamm Bowel Dis 20, 856–860 (2014). 24681654 10.1097/MIB.0000000000000016 PMC4077052

[62] Liu W. Intestinal epithelial vitamin D receptor signaling inhibits experimental colitis. J Clin Invest 123, 3983–3996 (2013). 23945234 10.1172/JCI65842 PMC3754241

[63] Nic Suibhne T., Cox G., Healy M., O’Morain C. & O’Sullivan M. Vitamin D deficiency in Crohn’s disease: Prevalence, risk factors and supplement use in an outpatient setting. J Crohns Colitis 6, 182–188 (2012). 22325172 10.1016/j.crohns.2011.08.002

[64] Jørgensen S. P. Clinical trial: vitamin D3 treatment in Crohn’s disease – a randomized double-blind placebo-controlled study. Alimentary Pharmacology & Therapeutics 32, 377–383 (2010). 20491740 10.1111/j.1365-2036.2010.04355.x

[65] Yang L. Therapeutic Effect of Vitamin D Supplementation in a Pilot Study of Crohn’s Patients. Clinical and Translational Gastroenterology 4, e33 (2013). 10.1038/ctg.2013.1 PMC3636524 23594800

[66] Hashash J. G., Elkins J., Lewis J. D. & Binion D. G. AGA Clinical Practice Update on Diet and Nutritional Therapies in Patients With Inflammatory Bowel Disease: Expert Review. Gastroenterology 166, 521–532 (2024). 38276922 10.1053/j.gastro.2023.11.303

[67] Houchen C. W., George R. J., Sturmoski M. A. & Cohn S. M. FGF-2 enhances intestinal stem cell survival and its expression is induced after radiation injury. American Journal of Physiology-Gastrointestinal and Liver Physiology 276, G249–G258 (1999). 10.1152/ajpgi.1999.276.1.G249 9887002

[68] Adas G. VEGF-A and FGF gene therapy accelerate healing of ischemic colonic anastomoses (experimental study). International Journal of Surgery 9, 467–471 (2011). 21642023 10.1016/j.ijsu.2011.05.002

[69] Dolivo D. M., Larson S. A. & Dominko T. Fibroblast Growth Factor 2 as an Antifibrotic: Antagonism of Myofibroblast Differentiation and Suppression of Pro-Fibrotic Gene Expression. Cytokine Growth Factor Rev 38, 49–58 (2017). 28967471 10.1016/j.cytogfr.2017.09.003 PMC5705586

[70] Wang P. Basic fibroblast growth factor reduces scar by inhibiting the differentiation of epidermal stem cells to myofibroblasts via the Notch1/Jagged1 pathway. Stem Cell Res Ther 8, 114 (2017). 28511663 10.1186/s13287-017-0549-7 PMC5434520

[71] Chen P.-Y., Qin L., Li G., Tellides G. & Simons M. Fibroblast growth factor (FGF) signaling regulates transforming growth factor beta (TGFβ)-dependent smooth muscle cell phenotype modulation. Sci Rep 6, 33407 (2016). 27634335 10.1038/srep33407 PMC5025753

[72] Sun C. Functions of exogenous FGF signals in regulation of fibroblast to myofibroblast differentiation and extracellular matrix protein expression. Open Biology 12, 210356 (2022). 36102060 10.1098/rsob.210356 PMC9471990

[73] Gallego-Muñoz P. Effects of TGFβ1, PDGF-BB, and bFGF, on human corneal fibroblasts proliferation and differentiation during stromal repair. Cytokine 96, 94–101 (2017). 28390267 10.1016/j.cyto.2017.03.011

[74] Strutz F. TGF-β1 induces proliferation in human renal fibroblasts via induction of basic fibroblast growth factor (FGF-2). Kidney International 59, 579–592 (2001). 11168939 10.1046/j.1523-1755.2001.059002579.x

[75] Graham M. F. Collagen content and types in the intestinal strictures of Crohn’s disease. Gastroenterology 94, 257–265 (1988). 3335305 10.1016/0016-5085(88)90411-8

[76] Bourgonje A. R. Serological biomarkers of type I, III and IV collagen turnover are associated with the presence and future progression of stricturing and penetrating Crohnʼs disease. Alimentary Pharmacology & Therapeutics 56, 675–693 (2022). 35661188 10.1111/apt.17063 PMC9544881

[77] Shimshoni E., Yablecovitch D., Baram L., Dotan I. & Sagi I. ECM remodelling in IBD: Innocent bystander or partner in crime? The emerging role of extracellular molecular events in sustaining intestinal inflammation. Gut 64, 367–372 (2015). 25416065 10.1136/gutjnl-2014-308048 PMC4345769

[78] Stallmach A., Schuppan D., Riese H. H., Matthes H. & Riecken E. O. Increased collagen type III synthesis by fibroblasts isolated from strictures of patients with Crohn’s disease. Gastroenterology 102, 1920–1929 (1992). 1587410 10.1016/0016-5085(92)90314-o

[79] Pakshir P. Dynamic fibroblast contractions attract remote macrophages in fibrillar collagen matrix. Nat Commun 10, 1850 (2019). 31015429 10.1038/s41467-019-09709-6 PMC6478854

[80] Lake S. P. & Barocas V. H. Mechanical and Structural Contribution of Non-Fibrillar Matrix in Uniaxial Tension: A Collagen-Agarose Co-Gel Model. Ann Biomed Eng 39, 1891–1903 (2011). 21416392 10.1007/s10439-011-0298-1 PMC3322425

[81] Li W., Chi N., Rathnayake R. A. C. & Wang R. Distinctive roles of fibrillar collagen I and collagen III in mediating fibroblast-matrix interaction: A nanoscopic study. Biochem Biophys Res Commun 560, 66–71 (2021). 33975247 10.1016/j.bbrc.2021.04.088 PMC8165026

[82] Carvalho S. N. Decreased collagen types I and IV, laminin, CK-19 and α-SMA expression after bone marrow cell transplantation in rats with liver fibrosis. Histochem Cell Biol 134, 493–502 (2010). 20963436 10.1007/s00418-010-0746-2

[83] Long A. M., Lee G., Demonbreun A. R. & McNally E. M. Extracellular matrix contribution to disease progression and dysfunction in myopathy. American Journal of Physiology-Cell Physiology 325, C1244–C1251 (2023). 37746696 10.1152/ajpcell.00182.2023 PMC10855263

[84] Lunde I. G., Rypdal K. B., Van Linthout S., Diez J. & González A. Myocardial fibrosis from the perspective of the extracellular matrix: Mechanisms to clinical impact. Matrix Biology 134, 1–22 (2024). 39214156 10.1016/j.matbio.2024.08.008

[85] Gao F. Basement Membrane Changes of Myofiber and Fibrosis in Sternocleidomastoid Muscle of Congenital Muscular Torticollis. Journal of Craniofacial Surgery 33, 2704 (2022). 36409848 10.1097/SCS.0000000000008781

[86] Friedman S. L. Hepatic Stellate Cells: Protean, Multifunctional, and Enigmatic Cells of the Liver. Physiological Reviews 88, 125–172 (2008). 18195085 10.1152/physrev.00013.2007 PMC2888531

[87] Ito T. & Kayama H. Roles of fibroblasts in the pathogenesis of inflammatory bowel diseases and IBD-associated fibrosis. International Immunology dxaf015 (2025) doi:10.1093/intimm/dxaf015. 40110813

[88] Simmons J. G., Pucilowska J. B., Keku T. O. & Lund P. K. IGF-I and TGF-β1 have distinct effects on phenotype and proliferation of intestinal fibroblasts. American Journal of Physiology-Gastrointestinal and Liver Physiology 283, G809–G818 (2002). 12181198 10.1152/ajpgi.00057.2002

[89] Sudi S., Suresh S. D., Kolli T. & Porras A. M. Trymethylamine-N-oxide, a gut-derived metabolite, induces myofibroblastic activation of valvular interstitial cells through endoplasmic reticulum stress. 2025.02.06.636980 Preprint at 10.1101/2025.02.06.636980 (2025).

[90] Stirling D. R. CellProfiler 4: improvements in speed, utility and usability. BMC Bioinformatics 22, 433 (2021). 34507520 10.1186/s12859-021-04344-9 PMC8431850

